# Effect of the COVID-19 pandemic on clinical characteristics and outcomes of adult pneumococcal meningitis patients – a Dutch prospective nationwide cohort study

**DOI:** 10.1007/s15010-024-02305-x

**Published:** 2024-06-03

**Authors:** Fabian D. Liechti, Merijn W. Bijlsma, Matthijs C. Brouwer, Diederik van de Beek

**Affiliations:** 1 Amsterdam UMC, Department of Neurology, Amsterdam Neuroscience, University of Amsterdam, Meibergdreef 9, PO Box 22660, Amsterdam, 1100DD Netherlands; 2 Department of General Internal Medicine, Inselspital, Bern University Hospital, University of Bern, Bern, Switzerland; 3 Department of Paediatrics, Amsterdam Neuroscience, Amsterdam UMC Location University of Amsterdam, Meibergdreef, Amsterdam, the Netherlands

**Keywords:** *Streptococcus pneumoniae*, Bacterial meningitis, Pneumococcal meningitis, COVID-19, Quality of health care, Alcoholism

## Abstract

**Purpose:**

To investigate clinical characteristics and outcomes of patients with pneumococcal meningitis during the COVID-19 pandemic.

**Methods:**

In a Dutch prospective cohort, risk factors and clinical characteristics of pneumococcal meningitis episodes occurring during the COVID-19 pandemic (starting March 2020) were compared with those from baseline and the time afterwards. Outcomes were compared with an age-adjusted logistic regression model.

**Results:**

We included 1,699 patients in 2006–2020, 50 patients in 2020*–*2021, and 182 patients in 2021–2023. After March 2020 relatively more alcoholism was reported (2006–2020, 6.1%; 2020–2021, 18%; 2021–2023, 9.7%; *P* = 0.002) and otitis–sinusitis was less frequently reported (2006–2020, 45%; 2020–2021, 22%; 2021–2023, 47%; *P* = 0.006). Other parameters, i.e. age, sex, symptom duration or initial C-reactive protein level, remained unaffected. Compared to baseline, lumbar punctures were more frequently delayed (on admission day, 2006–2020, 89%; 2020–2021, 74%; 2021–2022, 86%; *P* = 0.002) and outcomes were worse (‘good recovery’, 2020–2021, OR 0.5, 95% CI 0.3–0.8).

**Conclusion:**

During the COVID-19 pandemic, we observed worse outcomes in patients with pneumococcal meningitis. This may be explained by differing adherence to restrictions according to risk groups or by reduced health care quality.

**Supplementary Information:**

The online version contains supplementary material available at 10.1007/s15010-024-02305-x.

## Introduction

During the COVID-19 pandemic, pneumococcal meningitis incidence decreased [1, 2]; at the same time, healthcare systems were disrupted in preparation of admitting many COVID-19 patients, resulting in heterogeneous effects, including fewer non-COVID-19 hospitalisations [3, 4]. It is not known if and how the pandemic affected clinical characteristics and outcomes of specific diseases, e.g. by reduced hospital capacities or staying away from or not being sent to emergency departments [5]. In this observational study, we compared clinical characteristics and outcomes before and during the COVID-19 pandemic in a national clinical cohort of pneumococcal meningitis.

## Methods

We used data since 2006, from a national prospective cohort study of adults (≥ 16 years) with community-acquired bacterial meningitis, approved by the local ethical committee [6, 7]. Patients were recruited by notification from the Netherlands Reference Laboratory for Bacterial Meningitis or directly by physicians [7]. We defined cases as detecting *Streptococcus pneumoniae* by bacterial culture or polymerase chain reaction (PCR) in the cerebrospinal fluid (CSF) or a combination of CSF suggestive of bacterial meningitis (Spanos criteria, i.e. more than > 2,000 leukocytes per µL CSF, more than 1,180 polymorphonuclear leukocytes per µL CSF, CSF-serum glucose ratio < 0.23, CSF protein > 2.2 g/L, or CSF glucose < 1.9 mmol/L) and detecting pneumococci in blood samples by culture or in CSF samples by antigen testing [7].

We compared the first year of the pandemic, 2020–2021 (March 2020 to March 2021), with the baseline (2006–2020, March 2006 to March 2020) and did not consider shorter time intervals because of seasonality. To account for seasonal variation, we opted to use epidemiological years instead of calendar years. Because COVID-19 restrictions in the Netherlands were above the 25% margin between March 15, 2020 and March 14, 2022 according to the Oxford COVID-19 Government Response Tracker, we therefore decided to start an epidemiological year on 15 March and included 2020–2022 (March 2020 to March 2022) as sensitivity analysis. We used the Kruskal-Wallis test for continuous data and Chi-square and Fisher’s exact tests for categorical data.

Outcomes were assessed using the Glasgow Outcome Scale (1, ‘death’ to 5, ‘good recovery’) and compared using a logistic regression model (level 5 vs. levels 1 to 4) with age adjustment [7]. Mortality was compared using Kaplan-Meier estimates, based on survival data with censoring after hospital discharge. Missing values are shown.

## Results

We identified 1,699 cases in 2006–2020, 50 cases in 2020–2021, and 182 cases in 2021–2023 (Supplementary Figure S1), predominantly by pathogen detection in CSF samples by culture or PCR (2006–2020, *n* = 1,631, 96%; 2020–2021, *n* = 47, 94%; 2021–2023, *n* = 151, 83%) and more rarely by indicative CSF according to Spanos criteria (2006–2020, *n* = 65, 4%; 2020–2021, *n* = 3, 6%; 2021–2023, *n* = 31, 17%).

Age and sex distributions were similar across all three time periods (Table 1, Supplementary Figure S2). During the COVID-19 pandemic, the risk factor alcoholism was proportionately reported three times more frequently and otitis / sinusitis half as frequently. The clinical presentation, however, was similar, e.g. in terms of age, sex distribution, Glasgow Coma Scale score, symptom duration, or C-reactive protein level on admission. Differences in some variables, i.e. thrombocyte count or days to hospital discharge, reached statistical significance without a clinically relevant effect size. We found weak evidence for delayed treatment intensity in 2020–2021, i.e. fewer lumbar punctures were performed on the admission day, but no clear evidence for reductions in intensive care unit admissions (Table 1). The proportion of patients with pneumonia as complication did not change during the pandemic, while we observed more patients with persistent fever (body temperature of ⩾38 °C for > 10 days after the start of appropriate antimicrobial treatment) or seizures.

**Table 1:** Clinical characteristics, laboratory findings and outcomes of patients with pneumococcal meningitis (epidemiological years start March 15). (IQR, interquartile range; CSF, cerebrospinal fluid; ICU, intensive care unit)

Characteristic	*N*	2006–2020, *N* = 1,699	2020–2021, *N* = 50	2021–2023, *N* = 182	*P* value^1^
Age [years], Median (IQR)	1,931	62 (52–70)	64 (49–70)	62 (52–68)	0.71
Sex, *n* (%)	1,931				0.73
Female		857 (50)	23 (46)	88 (48)	
Male		842 (50)	27 (54)	94 (52)	
Immunosuppression, *n* (%)	1,931	464 (27)	18 (36)	59 (32)	0.15
Alcoholism, *n* (%)	1,923	103 (6.1)	9 (18)	17 (9.7)	0.002
History of cancer, *n* (%)	1,928	232 (14)	5 (10)	24 (13)	0.75
Diabetes mellitus, *n* (%)	1,917	241 (14)	11 (22)	29 (16)	0.27
History of splenectomy, *n* (%)	1,925	47 (2.8)	0 (0)	1 (0.6)	0.12
Otitis / sinusitis, *n* (%)	1,864	734 (45)	11 (22)	84 (47)	0.006
Pneumonia, *n* (%)	1,850	189 (12)	8 (16)	23 (13)	0.56
Symptoms < 24 h, *n* (%)	1,844	829 (51)	21 (45)	93 (53)	0.59
Glasgow Coma Scale score (range 3–15), Median (IQR)	1,921	10.0 (9.0–13.0)	10.0 (7.0–14.0)	10.0 (8.0–13.0)	0.33
Systolic blood pressure [mmHg], Median (IQR)	1,854	146 (130–165)	143 (129–161)	144 (127–160)	0.44
Heart rate [beats per minute], Median (IQR)	1,838	100 (85–115)	110 (89–129)	100 (88–115)	0.11
Leukocyte count [per µL], Median (IQR)	1,905	17 (12–23)	16 (12–25)	18 (11–25)	0.34
Thrombocyte count [per µL], Median (IQR)	1,825	199 (151–257)	203 (126–278)	224 (169–291)	0.002
C-reactive protein [mg/L], Median (IQR)	1,875	200 (94–317)	175 (57–293)	189 (97–292)	0.41
Blood culture, *n* (%)	1,758				0.40
Positive		1,302 (85)	39 (85)	155 (89)	
Negative		235 (15)	7 (15)	20 (11)	
CSF white cell count, Median (IQR)	1,841	2,297 (500–6,408)	1,480 (312–4,445)	2,905 (721–8,004)	0.052
CSF culture, *n* (%)	1,931				< 0.001
Positive		1,597 (94)	45 (90)	138 (76)	
Negative		102 (6.0)	5 (10)	44 (24)	
CSF Polymerase Chain Reaction positive for *S. pneumoniae*, *n* (%)	144	87 (40)	14 (78)	43 (86)	NA
Weisfelt score, Median (IQR)	284	0.70 (0.53–0.83)	0.66 (0.62–0.86)	0.59 (0.52–0.72)	0.34
Pretreatment with antibiotics, *n* (%)	1,884	172 (10)	1 (2.1)	16 (8.9)	0.14
Timing of lumbar puncture, *n* (%)	1,912				0.002
On admission day		1,498 (89)	37 (74)	156 (86)	
After admission day		182 (11)	13 (26)	26 (14)	
Intensive care, *n* (%)	1,931	1,102 (65)	28 (56)	110 (60)	0.23
Intensive care [days], Median (IQR)	361	4.0 (2.0–8.0)	5.0 (2.8–7.3)	4.0 (2.0–9.0)	0.84
Pneumonia as a complication, *n* (%)	1,799	292 (19)	8 (16)	33 (18)	0.92
Persistent fever^2^, *n* (%)	1,797	166 (11)	10 (20)	33 (19)	0.001
Seizure, *n* (%)	1,863	265 (16)	14 (28)	36 (20)	0.042
Days to death, Median (IQR)	331	6 (1–13)	7 (5–12)	9 (3–16)	0.64
Days to hospital discharge, Median (IQR)	1,567	15 (12–22)	15 (13–22)	14 (11–19)	0.006
Glasgow Outcome Scale, *n* (%)	1,931				n/a
Dead		301 (18)	15 (30)	23 (13)	
Vegetative survival		3 (0.2)	0 (0)	1 (0.5)	
Severely disabled		86 (5.1)	3 (6.0)	4 (2.2)	
Moderately disabled		306 (18)	12 (24)	38 (21)	
Good recovery		1,003 (59)	20 (40)	116 (64)	

^1^Kruskal-Wallis rank sum test; Pearson’s Chi-squared test; Fisher’s exact test

^2^Body temperature of ⩾38 °C for > 10 days after the start of appropriate antimicrobial treatment

**Fig. 1:**
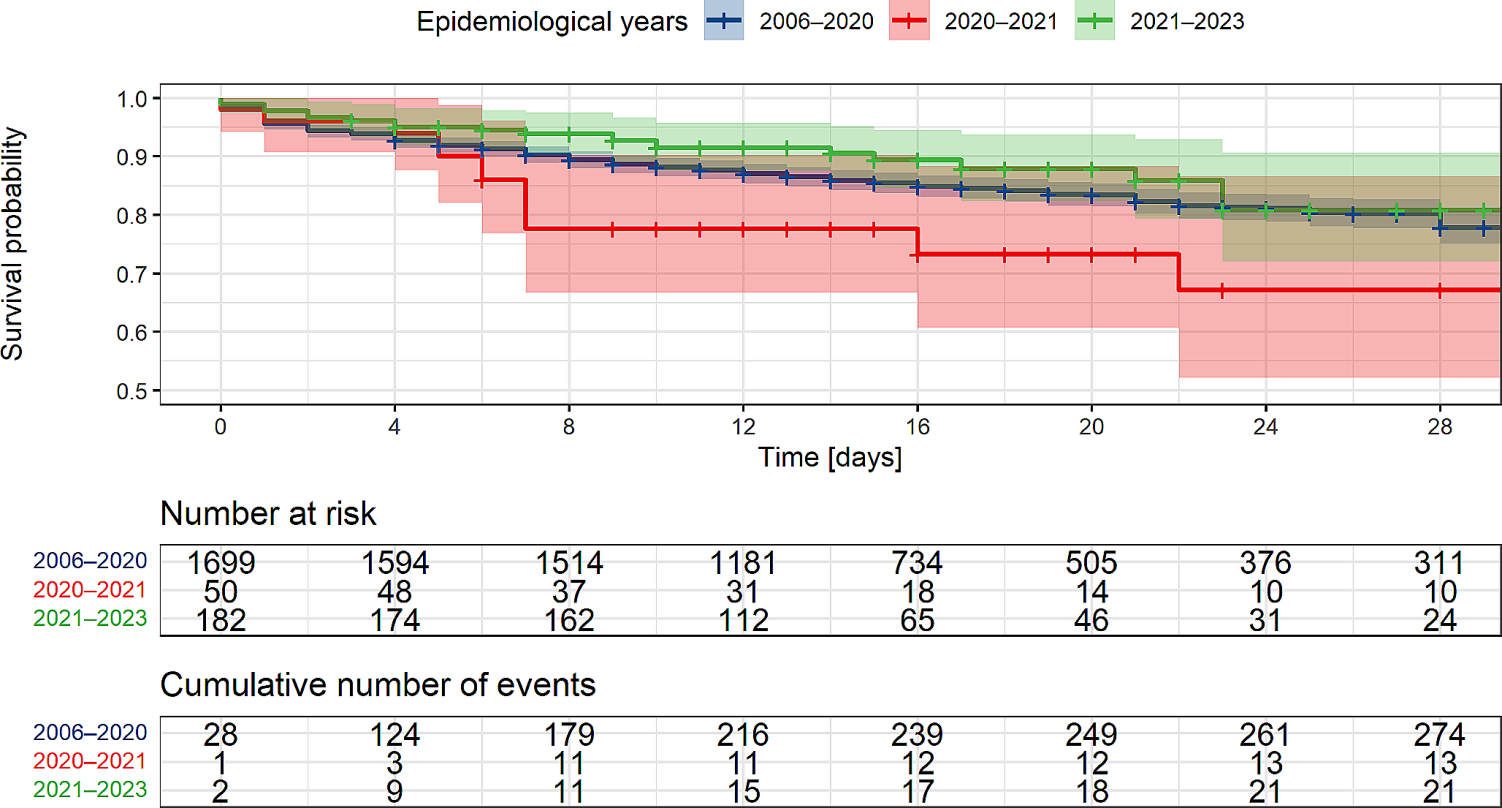
Kaplan-Meier survival curve including 95% confidence intervals comparing survival of pneumococcal meningitis patients in 2006–2020 with 2020–2021 and 2021–2023 (log-rank test, *P* = 0.2; Peto & Peto modified Gehan-Wilcoxon test, *P* = 0.2). Follow-up times were censored at 28 days or discharge from hospital

Distribution of initial Glasgow Coma Scale scores was similar across time periods (Supplementary Figure S3) Outcomes of patients hospitalized were worse in 2020–2021 (age-adjusted OR for ‘good recovery’ = 0.5, 95% CI 0.3–0.8, *P* = 0.011), but not in 2021–2023 (OR 1.1, 95% CI 0.8–1.6, *P* = 0.44), compared to 2006–2020 (Supplementary Figure S4). Accordingly, in-hospital survival was worse in 2020–2021 (*P* value 0.02, Fig. 1) with lower 14-day survival during the COVID-19 pandemic (2006–2020, 86%, 95% CI 84–88%; 2020–2021, 78%, 95% CI 67–90%; 2021–2023, 91%, 95% CI 86–95%).

In the sensitivity analysis, comparing the first two years of the COVID-19 pandemic (2020–2022) with baseline (2006–2020) and the time afterwards (2022–2023), results were confirmed with higher proportion of alcoholism and later performance of lumbar puncture (Supplementary Table S1). We did not find a lower proportion of patients with sinusitis or otitis, but fewer patients with a history of cancer, and fewer admissions to intensive care, while outcomes were comparable to baseline when including the first two years of the pandemic (Supplementary Figure S5).

## Discussion

During the COVID-19 pandemic, patients with pneumococcal meningitis differed in terms of comorbidities and hospital care: Proportionately more meningitis patients reported alcoholism, but because the overall meningitis incidence was considerably lower during the COVID-19 pandemic, the absolute number of cases with meningitis and concomitant alcoholism remained stable. In the same time period, fewer patients had concomitant otitis / sinusitis and lumbar punctures were performed later. We found strong evidence for worse outcomes during the first year of the COVID-19 pandemic – an effect not manifest when considering outcomes of the first two years.

Several factors could have biased this observation of worse outcomes. First, pre-hospital decisions to reduce treatment intensity and avoiding hospitalisation, e.g. in frail people, would have led to an underestimation of severe outcomes and mortality. Second, impaired pre-clinical care or late presentation, would have led to opposite effects, similar to our findings. Moreover, symptom duration or antibiotic treatment before admission did not increase during the COVID-19 pandemic. Third, worse outcomes may be explained by a higher proportion of patients presenting with a high risk for adverse outcome per se, namely advanced age, absence of otitis / sinusitis, alcoholism, tachycardia, lower score on the Glasgow Coma Scale, a low CSF white-cell count, a positive blood culture, and a high serum C-reactive protein concentration [7]. Indeed, otitis / sinusitis were reported less frequently and alcoholism was reported relatively more frequently, thus potentially explaining worse outcomes in 2020–2021. A higher association of otitis / sinusitis with influenza virus compared to COVID-19, which was initially mostly associated with pneumonia, could explain why we identified fewer patients with otitis / sinusitis in 2020–2021, an observation compatible with the lower incidence of otitis during the pandemic in the general population [8–10]. The lower proportion of patients with otitis / sinusitis could also explain the lower proportion of patients receiving antibiotics before hospital admission in 2020–2021 because patients with otitis / sinusitis as a distant focus of infection more commonly present with antibiotic treatment through their GP compared to patients without otitis / sinusitis. Alternatively a presumed diagnosis of COVID-19 as the cause of the symptoms could have resulted in lower pretreatment rates. Alcoholism is a risk factor for invasive pneumococcal disease and worse outcome in meningitis [11–13]. During the COVID-19 pandemic, alcohol-associated mortality was reported to be higher in many countries, although not in the Netherlands, and problematic alcohol consumption increased [14–16]. Because in our study, the absolute number of patients with meningitis and concomitant alcoholism was similar between the different time periods, we hypothesize that the pandemic and all social changes that the pandemic entailed, had a smaller or no effect on behaviour that is associated with pneumococcal meningitis risk in people with reported alcoholism. This is different from the risk of pneumococcal meningitis for the general population in the Netherlands [2]. However, data that behaviour changes during the pandemic differed in specific sub-groups of the population e.g. in those with immunosuppression or alcoholism, are limited while self-reported alcoholism could also be an indicator of socio-economically disadvantaged persons [17–20]. Fourth, in our cohort we found more patients with persistent fever and seizures, which could not only be attributed to more severe clinical presentation at admission, but also reduced quality of care. More in-hospital complications – but not increasing mortality – was earlier described in non-COVID-19 patients during the first year of the pandemic [3]. Similarly, worse outcomes in 2020*–*2021 may also be related to hospital-related factors. Indeed, we observed delays in performing lumbar punctures – explaining the lower proportion of positive cultures – and possibly lower admission rates for intensive care. This may indicate that the health care system was ‘under stress’ with limited service availability or deliberate early reduction in treatment intensity in some cases and thus higher mortality during the COVID-19 pandemic. Survival rates after the first week of the infection differed between the periods. Some patients who are comatose on admission still fully recover [21]. However, we did not find any evidence that during the COVID-19 pandemic more patients with low Glasgow Coma scale scores on admission died.

Our well-established national cohort was firmly maintained throughout the COVID-19 pandemic allowing solid comparisons. However, other confounders could have affected our results despite adjusting for age in the model used, and the total number of cases available for analysis during the COVID-19 pandemic was small, thus limiting further adjustments. The sensitivity analysis covered the time interval until March 15, 2022, when contingency measures were officially reduced; however, since their inception in March 2020, the measures’ strength varied and willingness to follow recommendations probably waned. Also, outcomes were available only until hospital discharge; however, we used censored data in the survival analysis.

In conclusion, some people at high risk for pneumococcal meningitis had reduced their risk (i.e. splenectomized and cancer patients), while others had not (people with alcoholism). This, together with potentially reduced performance of the health-care system, would explain the worse outcomes during the COVID-19 pandemic, despite similar baseline characteristics.

## Electronic supplementary material

Below is the link to the electronic supplementary material.

Supplementary Material 1

## Data Availability

Although data protection regulations in the Netherlands do not allow sharing of individual participant data, datasets with selected aggregated data are available upon reasonable request. Individuals who request data will be asked to sign a data access agreement.
