# Supplementary material for: Effect of the COVID-19 pandemic on clinical characteristics and outcomes of adult pneumococcal meningitis patients – a Dutch prospective nationwide cohort study
Source: Infection. 2024 Jun 3;52(5):1657–62. doi: 10.1007/s15010-024-02305-x (PMC11499351; doi:10.1007/s15010-024-02305-x)
Supplement: Supplementary file 1 — Supplementary Material 1 [file 15010_2024_2305_MOESM1_ESM.pdf]

# Supplement

Liechti FD, Bijlsma MW, Brouwer MC, van de Beek D

**Supplementary Table S1.** Clinical characteristics of patients with pneumococcal meningitis (sensitivity analysis). (IQR, interquartile range; CSF, cerebrospinal fluid; ICU, intensive care unit) ... 2

**Supplementary Figure S1.** Flow-chart. Patients were reported by the National Reference Laboratory for Bacterial Meningitis to the MeninGene cohort study team or directly recruited by physicians. (CSF, cerebrospinal fluid; PCR, polymerase chain reaction; Spanos criteria, more than > 2,000 leukocytes per µL CSF, more than 1,180 polymorphonuclear leukocytes per µL CSF, CSF-serum glucose ratio < 0.23, CSF protein > 2.2 g/L, or CSF glucose < 1.9 mmol/L)........................................ 4

**Supplementary Figure S2.** Age of patients with pneumococcal meningitis stratified by epidemiological years and in-hospital death........................................................................................ 5

**Supplementary Figure S3.** Distribution of Glasgow Coma Scale scores on admission with cases presented as densities to allow easier comparison. ................................................................................ 6

**Supplementary Figure S4.** Distribution of Glasgow Outcome Scale scores of pneumococcal meningitis patients in 2006–2020, 2020–2021 and 2020–2022............................................................. 7

**Supplementary Figure S5.** Kaplan-Meier survival curve including 95% confidence intervals of pneumococcal meningitis patients in 2006–2020, 2020–2022 and 2022–2023 (sensitivity analysis; log-rank test, p = 0.5; Peto & Peto modified Gehan-Wilcoxon test p = 0.5). Follow-up times were censored at 28 days or discharge from hospital. ..................................................................... 8

## Supplementary tables

**Supplementary Table S1.** Clinical characteristics of patients with pneumococcal meningitis (sensitivity analysis). (IQR, interquartile range; CSF, cerebrospinal fluid; ICU, intensive care unit)

| Characteristic | N | 2006–2020, N = 1,699 | 2020–2022, N = 133 | 2022–2023, N = 99 | p-value[1] |
|---|---|---|---|---|---|
| **Age [years], Median (IQR)** | 1,931 | 62 (52–70) | 61 (48–68) | 64 (56–69) | 0.34 |
| **Sex, n (%)** | 1,931 | | | | 0.75 |
| Female | | 857 (50) | 63 (47) | 48 (48) | |
| Male | | 842 (50) | 70 (53) | 51 (52) | |
| **Predisposition** | | | | | |
| Immunosuppression, n (%) | 1,931 | 464 (27) | 42 (32) | 35 (35) | 0.14 |
| Alcoholism, n (%) | 1,923 | 103 (6.1) | 16 (12) | 10 (11) | 0.008 |
| History of cancer, n (%) | 1,928 | 232 (14) | 10 (7.5) | 19 (19) | 0.030 |
| Diabetes mellitus, n (%) | 1,917 | 241 (14) | 24 (18) | 16 (16) | 0.42 |
| History of splenectomy, n (%) | 1,925 | 47 (2.8) | 0 (0) | 1 (1.0) | 0.071 |
| Otitis–sinusitis, n (%) | 1,864 | 734 (45) | 56 (43) | 39 (40) | 0.61 |
| Pneumonia, n (%) | 1,850 | 189 (12) | 18 (14) | 13 (13) | 0.68 |
| **Symptoms < 24h , n (%)** | 1,844 | 829 (51) | 62 (49) | 52 (55) | 0.68 |
| **Glasgow Coma Scale score (range 3–15), Median (IQR)** | 1,921 | 10.0 (9.0–13.0) | 10.0 (8.0–14.0) | 9.0 (7.0–12.0) | 0.014 |
| **Systolic blood pressure [mmHg], Median (IQR)** | 1,854 | 146 (130–165) | 144 (127–160) | 146 (130–163) | 0.43 |
| **Heart rate [beats per minute], Median (IQR)** | 1,838 | 100 (85–115) | 105 (90–121) | 98 (84–114) | 0.073 |
| **Leukocyte count [per µL], Median (IQR)** | 1,905 | 17 (12–23) | 17 (12–24) | 18 (11–26) | 0.44 |
| **Thrombocyte count [per µL], Median (IQR)** | 1,825 | 199 (151–257) | 221 (172–284) | 213 (153–293) | 0.006 |
| **C-reactive protein [mg/L], Median (IQR)** | 1,875 | 200 (94–317) | 161 (63–284) | 209 (128–299) | 0.074 |
| **Blood culture, n (%)** | 1,758 | | | | 0.48 |
| Positive | | 1,302 (85) | 112 (88) | 82 (87) | |
| Negative | | 235 (15) | 15 (12) | 12 (13) | |
| **CSF white cell count, Median (IQR)** | 1,841 | 2,297 (500–6,408) | 2,652 (722–7,147) | 2,453 (442–7,690) | 0.51 |
| **CSF culture, n (%)** | 1,931 | | | | <0.001 |
| Positive | | 1,597 (94) | 103 (77) | 80 (81) | |
| Negative | | 102 (6.0) | 30 (23) | 19 (19) | |
| **CSF Polymerase Chain Reaction positive for *S. pneumoniae*, n (%)** | 144 | 87 (40) | 30 (81) | 27 (87) | <0.001 |

| | | | | | |
|---|---|---|---|---|---|
| **Weisfelt score, Median (IQR)** | 284 | 0.70 (0.53–0.83) | 0.64 (0.53–0.78) | 0.65 (0.55–0.75) | 0.56 |
| **Pretreatment with antibiotics, n (%)** | 1,884 | 172 (10) | 9 (6.9) | 8 (8.2) | 0.37 |
| **Timing of lumbar puncture, n (%)** | 1,912 | | | | 0.002 |
| On admission day | | 1,498 (89) | 105 (79) | 88 (89) | |
| After admission day | | 182 (11) | 28 (21) | 11 (11) | |
| **Intensive care, n (%)** | 1,931 | 1,102 (65) | 73 (55) | 65 (66) | 0.066 |
| **Intensive care [days], Median (IQR)** | 361 | 4.0 (2.0–8.0) | 4.0 (2.0–8.0) | 4.0 (2.0–10.0) | 0.51 |
| **Complication - Pneumonia, n (%)** | 1,799 | 292 (19) | 19 (15) | 22 (22) | 0.33 |
| **Persisting fever, n (%)** | 1,797 | 166 (11) | 21 (16) | 22 (23) | <0.001 |
| **Seizures, n (%)** | 1,863 | 265 (16) | 32 (24) | 18 (19) | 0.049 |
| **Days to death, Median (IQR)** | 331 | 6 (1–13) | 7 (5–11) | 10 (2–23) | 0.61 |
| **Days to hospital discharge, Median (IQR)** | 1,567 | 15 (12–22) | 14 (11–18) | 15 (11–21) | 0.002 |
| **Glasgow Outcome Scale, n (%)** | 1,931 | | | | |
| Dead | | 301 (18) | 24 (18) | 14 (14) | |
| Vegetative survival | | 3 (0.2) | 1 (0.8) | 0 (0) | |
| Severely disabled | | 86 (5.1) | 6 (4.5) | 1 (1.0) | |
| Moderately disabled | | 306 (18) | 24 (18) | 26 (26) | |
| Good recovery | | 1,003 (59) | 78 (59) | 58 (59) | |

[1]Kruskal-Wallis rank sum test; Pearson's Chi-squared test; Fisher's exact test

# Supplementary figures

**Supplementary Figure S1.** Flow-chart. Patients were reported by the National Reference Laboratory for Bacterial Meningitis to the MeninGene cohort study team or directly recruited by physicians. (CSF, cerebrospinal fluid; PCR, polymerase chain reaction; Spanos criteria, more than > 2,000 leukocytes per µL CSF, more than 1,180 polymorphonuclear leukocytes per µL CSF, CSF-serum glucose ratio < 0.23, CSF protein > 2.2 g/L, or CSF glucose < 1.9 mmol/L)

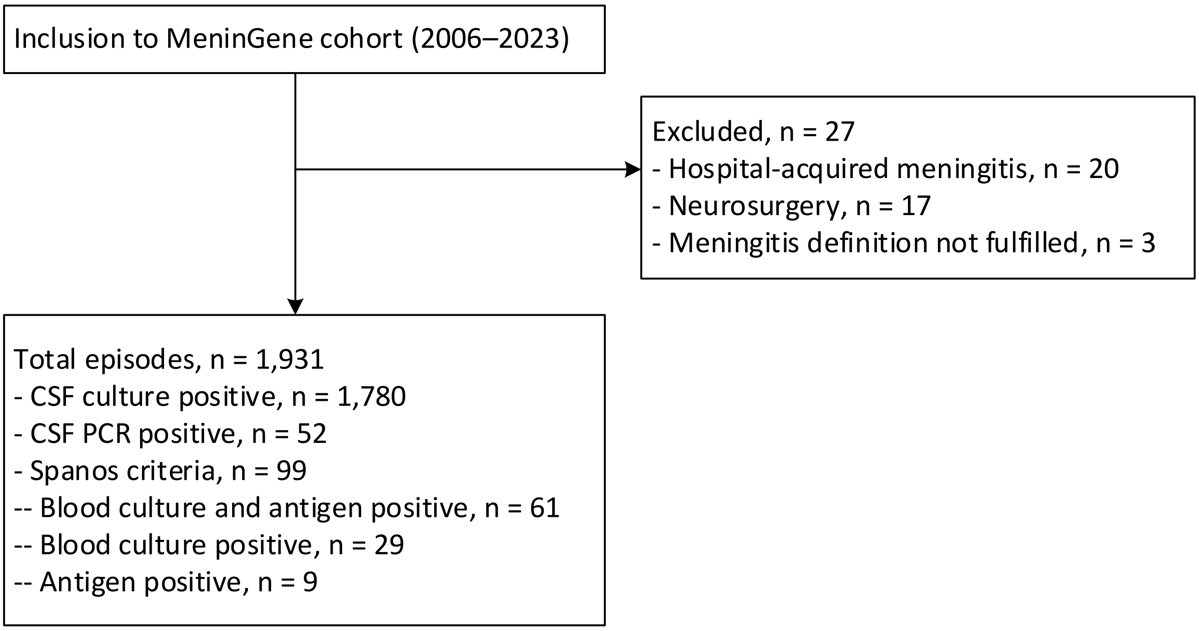

**Supplementary Figure S2.** Age of patients with pneumococcal meningitis stratified by epidemiological years and in-hospital death.

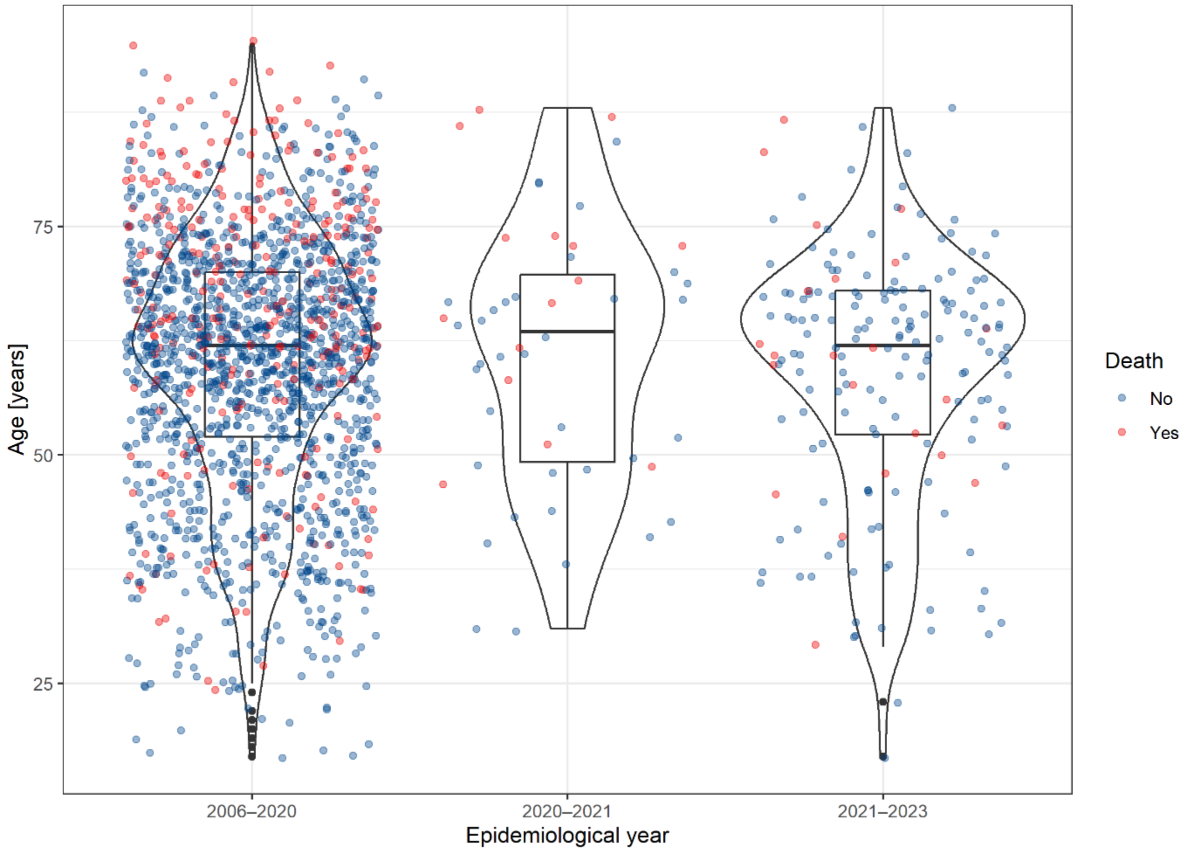

**Supplementary Figure S3.** Distribution of Glasgow Coma Scale scores on admission with cases presented as densities to allow easier comparison.

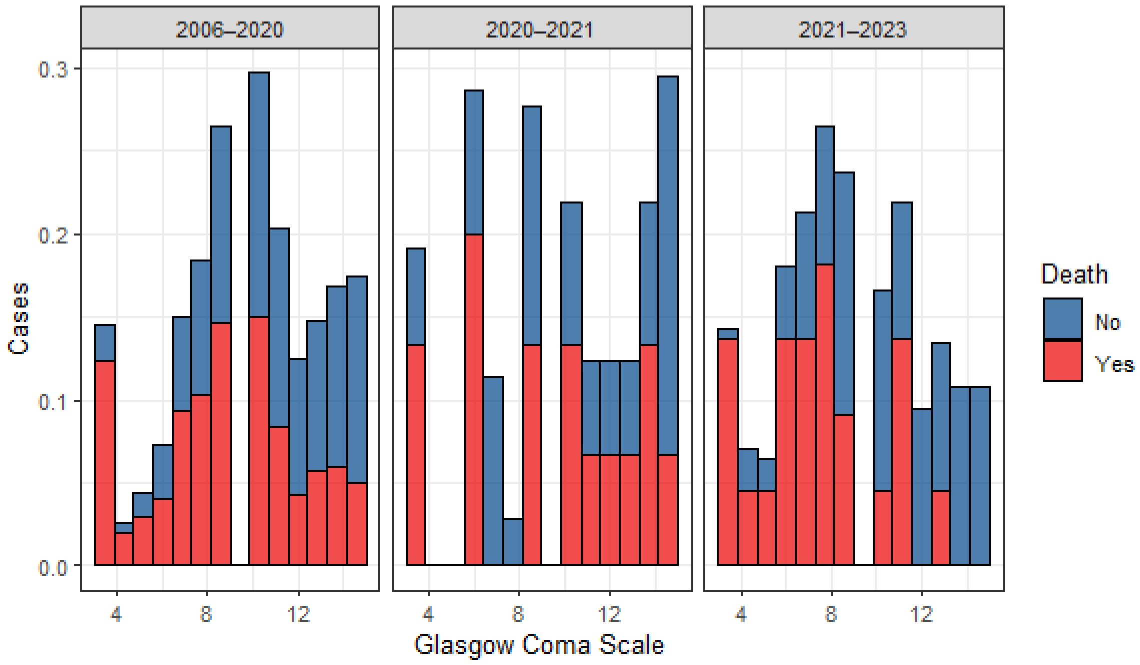

**Supplementary Figure S4.** Distribution of Glasgow Outcome Scale scores of pneumococcal meningitis patients in 2006–2020, 2020–2021 and 2020–2022.

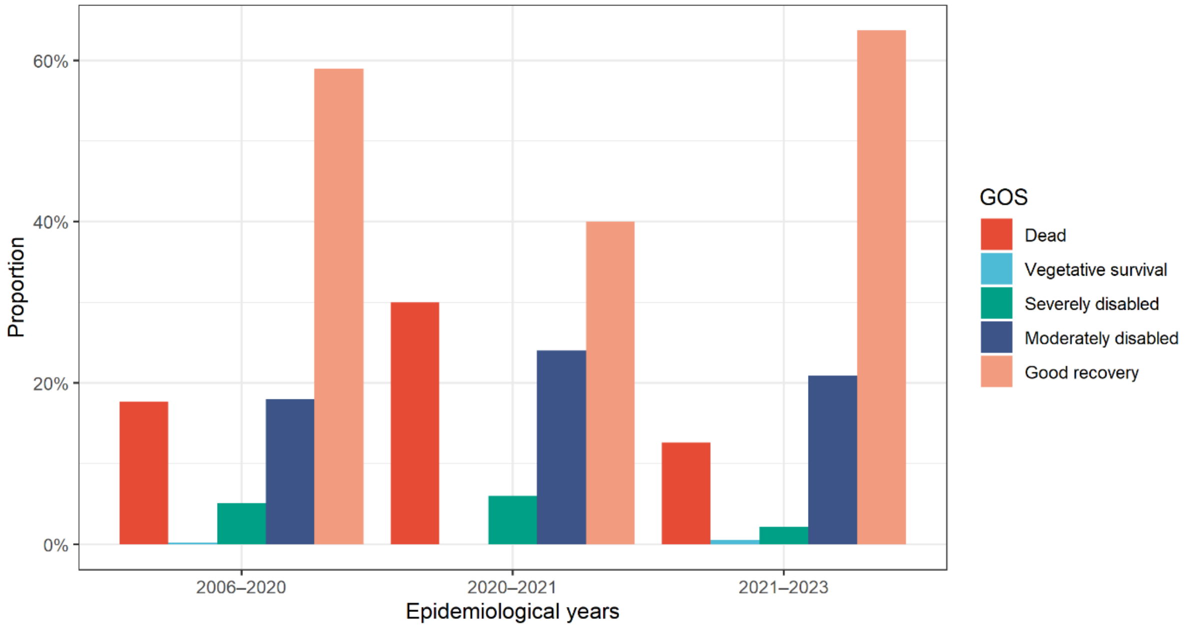

**Supplementary Figure S5.** Kaplan-Meier survival curve including 95% confidence intervals of pneumococcal meningitis patients in 2006–2020, 2020–2022 and 2022–2023 (sensitivity analysis; log-rank test, p = 0.5; Peto & Peto modified Gehan-Wilcoxon test p = 0.5). Follow-up times were censored at 28 days or discharge from hospital.

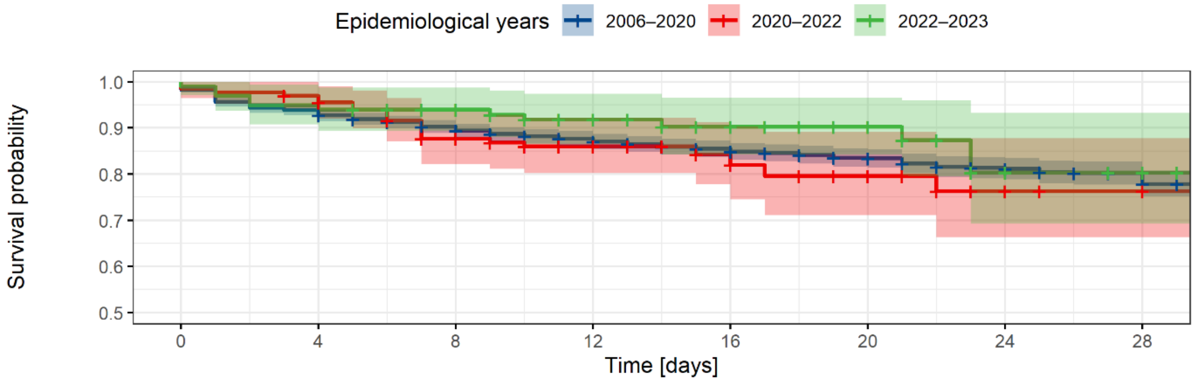


Number at risk

| | | | | | | | | |
|---|---|---|---|---|---|---|---|---|
| 2006–2020 | 1699 | 1594 | 1514 | 1181 | 734 | 505 | 376 | 311 |
| 2020–2022 | 133 | 128 | 109 | 76 | 37 | 28 | 19 | 14 |
| 2022–2023 | 99 | 94 | 90 | 67 | 46 | 32 | 22 | 20 |

Cumulative number of events

| | | | | | | | | |
|---|---|---|---|---|---|---|---|---|
| 2006–2020 | 28 | 124 | 179 | 216 | 239 | 249 | 261 | 274 |
| 2020–2022 | 2 | 6 | 16 | 18 | 20 | 21 | 22 | 22 |
| 2022–2023 | 1 | 6 | 6 | 8 | 9 | 9 | 12 | 12 |
